# 464. Structural Vulnerability among Patients with HIV and SARS-CoV-2 Coinfection: Descriptive Case Series from the U.S. Midwest

**DOI:** 10.1093/ofid/ofab466.663

**Published:** 2021-12-04

**Authors:** Natasha E Hongsermeier-Graves, Rohan Khazanchi, Nada Fadul

**Affiliations:** 1 University of Nebraska Medical Center, Omaha, Nebraska; 2 College of Medicine, University of Nebraska Medical Center, Omaha, NE

## Abstract

**Background:**

It is well known that the HIV epidemic and COVID-19 pandemic have both disproportionately harmed marginalized minority and immigrant communities in the United States. The risk factors associated with disease incidence and outcomes reaffirm that *structural vulnerabilities*—sociopolitically imposed risk factors like discrimination, legal status, poverty, and beyond which impact a patient’s opportunity to achieve optimal health—play a key role in facilitating the inequitable harms of COVID-19 and HIV alike. This study explores the role of structural forces in increasing the risk of SARS-CoV-2 coinfection among people with HIV (PWH).

**Methods:**

We performed a retrospective chart review of PWH receiving care at the University of Nebraska Medical Center HIV clinic in Omaha, Nebraska, to collect patient demographics, comorbidities, HIV outcomes, and COVID-19 outcomes for 37 patients with HIV and SARS-CoV-2 coinfection as of August 27, 2020. As a comparison group, we obtained demographic data from a registry of all patients seen at the HIV clinic. We used R Statistical Software to perform descriptive statistical analysis.

**Results:**

Relative to our overall HIV clinic population, over twice as many Hispanic patients (35.1% vs. 16.0%), three times as many undocumented patients (13.5% vs. 4.2%), and four times as many refugee patients (16.2% vs. 4.0%) had COVID-19. The majority (67.6%) of coinfected patients reported working in “essential” jobs during the pandemic. Thirty-four of the 37 people with HIV and COVID-19 (PWHC) had at least one comorbidity, including increased BMI (83.7%), hypertension (64.9%), or hyperlipidemia (48.6%). All 37 PWHC remained alive as of October 4, 2020.

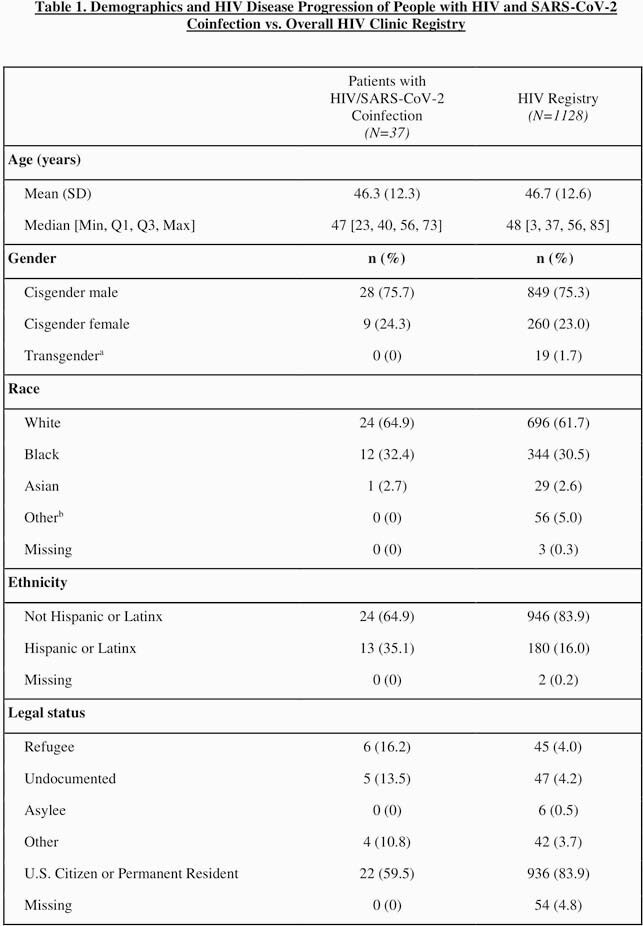

Demographics and HIV Disease Progression of People with HIV and SARS-CoV-2 Coinfection vs. Overall HIV Clinic Registry

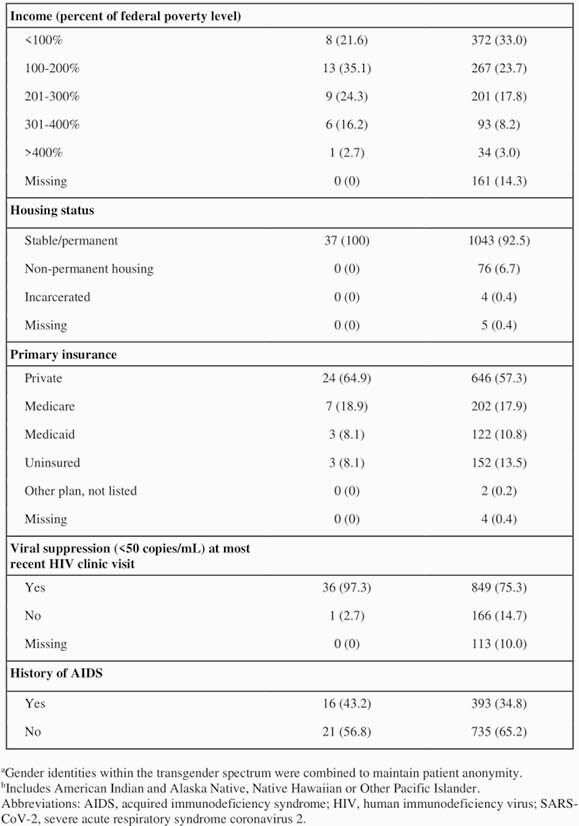

Demographics and HIV Disease Progression of People with HIV and SARS-CoV-2 Coinfection vs. Overall HIV Clinic Registry (continued)

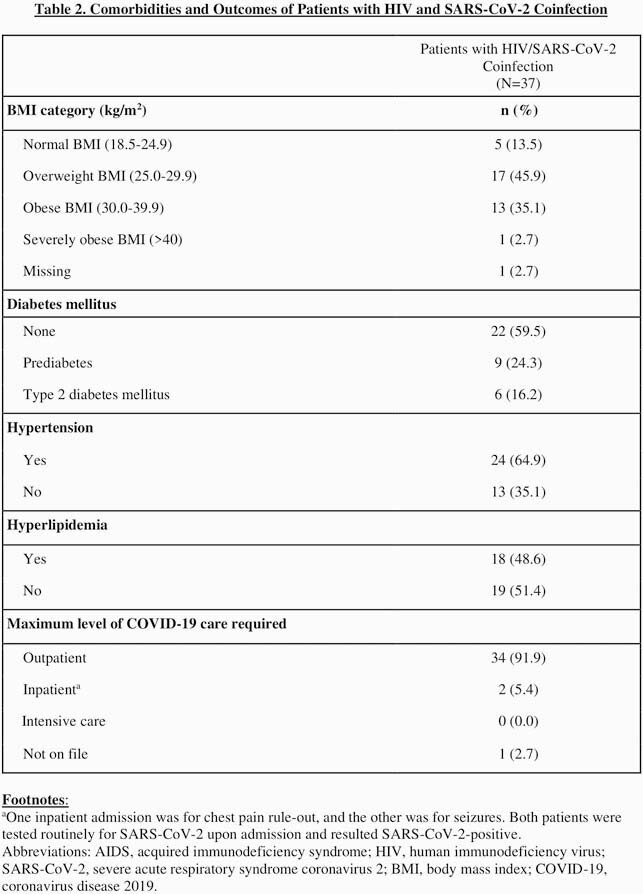

**Conclusion:**

The disproportionate burden of SARS-CoV-2 coinfection on Hispanic, undocumented, and refugee PWH may be a product of structural vulnerabilities contributing to greater risk of exposure. Although all 37 PWHC had well-controlled HIV and relatively mild COVID-19 courses, the broader theme of disproportionate COVID-19 incidence among vulnerable sub-populations of people with HIV reaffirms the importance of structural interventions to mitigate current and downstream harms.

**Disclosures:**

**All Authors**: No reported disclosures

